# BARRIERS AND FACILITATORS TO INTEGRATED SUBSTANCE USE AND HIV CARE: ADMINISTRATOR AND PROVIDER PERSPECTIVES

**DOI:** 10.1093/geroni/igad104.0380

**Published:** 2023-12-21

**Authors:** Alexis Bender, Kiera Chan, Riley Hunt, Molly Perkins

**Affiliations:** Emory University, Atlanta, Georgia, United States; Emory University, Atlanta, Georgia, United States; Emory University, Atlanta, Georgia, United States; Emory University, Atlanta, Georgia, United States

## Abstract

Since the beginning of the HIV epidemic, HIV and substance use have been intricately connected. Substance use may cause additional damage to the brain, higher viral loads, low medication adherence, and higher AIDS-related deaths. A potential area to improve access to care and treatment for people aging with HIV and substance use is through integrated care. Yet little is known about the barriers and facilitators to integrated care, especially in practices serving vulnerable populations, such as older adults or PLWH in rural areas. We conducted in-depth interviews with 21 providers (e.g., Physicians, APPs, RNs) and 15 administrators (e.g., clinic managers, care coordinators) across Georgia between January 2021 and November 2022. Interviews explored perceptions about clinic integration and desire, or lack of desire, to increase integration. Data were analyzed using inductive and deductive thematic analysis guided by the Consolidated Framework for Implementation Research. Both groups perceived important benefits to integrated care, especially for older or more vulnerable populations, but the definitions varied across respondents. Providers and administrators agreed that integrated care provided greater access to care with less interruption to patients’ lives. Barriers to increasing integration included, but were not limited to, physical space, staffing and budgetary constraints, and limited opportunities for collaboration. Most providers, except those in practices with the highest levels of integration, experienced barriers to getting patients into adequate substance use treatment when needed. Integrated care is vital to treating older adults and vulnerable individuals living with HIV, especially those with co-occurring substance use disorders.

